# Supplementary material for: Genetic association of ANRIL with susceptibility to Ischemic stroke: A comprehensive meta-analysis
Source: PLoS One. 2022 Jun 2;17(6):e0263459. doi: 10.1371/journal.pone.0263459 (PMC9162336; doi:10.1371/journal.pone.0263459)
Supplement: S3 Appendix — (DOCX) [file pone.0263459.s003.docx]

| A total of 856 records were identified by searching in English and Chinese databases at first. After 747 records were deleted through screening by title and/or Abstract , 115 full texts remained to be further selected. | |
| --- | --- |
| Case report=1 | 1. Voronovich ZA. Restrictive cerebral cortical venopathy: A new clinicopathological entity. 2019. |
| Family studies=1 | 2. Niemiec P. The rs10757278 Polymorphism of the 9p21.3 Locus in Children with Arterial Ischemic Stroke: A Family-Based and Case-Control Study. 2017. |
| Animal studies=6 | 3. Zhong W. lncRNA ANRIL Ameliorates Oxygen and Glucose Deprivation (OGD) Induced Injury in Neuron Cells via miR‑199a‑5p/CAV‑1 Axis. 2020;  4. Lei JJ. Long noncoding RNA CDKN2B-AS1 interacts with transcription factor BCL11A to regulate progression of cerebral infarction through mediating MAP4K1 transcription. 2019;  5. Zhao JH. Influence of lncRNA ANRIL on neuronal apoptosis in rats with cerebral infarction by regulating the NF-κB signaling pathway. 2019;  6. Liu B. LncRNA ANRIL protects against oxygen and glucose deprivation (OGD)-induced injury in PC-12 cells: potential role in ischaemic stroke. 2019;  7. Zhang B. Overexpression of lncRNA ANRIL up-regulates VEGF expression and promotes angiogenesis of diabetes mellitus combined with cerebral infarction by activating NF-κB signaling pathway in a rat model. 2017;  8. Jiang XL. LncRNA ANRIL interfered with miR-153 and mediated nuclear translocation of NF-κB P65 to induce brain tissue injury, apoptosis and oxidative stress in mice with cerebral ischemia reperfusion (In Chinese). 2021. |
| Meta-analysis=8 | 9. Tan C. Effects of ANRIL variants on the risk of ischemic stroke: a meta-analysis. 2019;  10. Traylor M. Genetic risk factors for ischaemic stroke and its subtypes (the METASTROKE Collaboration): a meta-analysis of genome-wide association studies. 2012;  11. Chen JX. Genetic variants on chromosome 9p21 confer risks of cerebral infarction in the Chinese population: a meta-analysis. 2019;  12. Ni X. Association between 9p21 Genomic Markers and Ischemic Stroke Risk: Evidence Based on 21 Studies. 2014;  13. Guo J. Association between 9p21.3 genomic markers and coronary artery disease in East Asians: a meta-analysis involving 9,813 cases and 10,710 controls. 2013; 14. He FY. Long Chain Non-coding RNA-ANRIL rs2383207 Polymorphism and Risk of Ischemic Stroke: a Meta Analysis (In Chinese). 2018; 15. Liu W. Association between rs10757274 in chromosome 9p21 and ischemic stroke: A comprehensive Meta-analysis (In Chinese). 2016;  16. Liu W. Association between the Single Nucleotide Polymorphisms on Chromosome 9p21 and Ischemic Stroke: A Comprehensive Meta-Analysis (In Chinese). 2016. |
| Reviews=13 | 17. Bao MH. Long non-coding RNAs in ischemic stroke. 2018;  18. Kong YH. ANRIL: A IncRNA at the CDKN2A/B Locus With Roles in Cancer and Metabolic Disease. 2018;  19. Kumar S. Role of flow-sensitive microRNAs and long noncoding RNAs in vascular dysfunction and atherosclerosis. 2018;  20. Gupta SC. Long non-coding RNAs and nuclear factor-κ κB crosstalk in cancer and other human diseases. 2020;  21. Song CY. CDKN2B-AS1: An Indispensable Long Non-coding RNA in Multiple Diseases. 2020;  22. Holdt LM. Long Noncoding RNA ANRIL: Lnc-ing Genetic Variation at the Chromosome 9p21 Locus to Molecular Mechanisms of Atherosclerosis. 2018;  23. Popov N. Epigenetic regulation of the INK4b-ARF-INK4a Locus. 2016;  24. Florian IA. Deciphering the vascular labyrinth: role of microRNAs and candidate gene SNPs in brain AVM development – literature review. 2020;  25. Holdt LM. Recent Studies of the Human Chromosome 9p21 Locus, Which Is Associated With Atherosclerosis in Human Populations. 2012;  26. Yamada Y. Proinflammatory Gene Polymorphisms and Ischemic Stroke. 2008;  27. Palomaki GE. Use of genomic profiling to assess risk for cardiovascular disease and identify individualized prevention strategies—A targeted evidence-based review. 2010;  28. Wang Y. Advance in the research of long non-coding RNA and ischemic stroke (In Chinese). 2020;  29. Zhong CQ. Research Progress in Correlation between 9p21 Polymorphism and Stroke (In Chinese). 2020. |
| Just one study of SNP  involved=1 | 30. Fathy N. Long Noncoding RNAs MALAT1 and ANRIL Gene Variants and the Risk of Cerebral Ischemic Stroke: An Association Study. 2021. |
| Controls containing TIA=2 | 31. Plant SR. Exploration of a Hypothesized Independent Association of a Common 9p21.3 Gene Variant and Ischemic Stroke in Patients with and without Angiographic Coronary Artery Disease. 2010;  32. Lemmens R. Variant on 9p21 strongly associates with coronary heart disease, but lacks association with common stroke. 2009. |
| Multiple studies from same  author/possible duplicated  study=3 | 33. Chen XL. The effect of ncRNA ANRIL on atherothrombotic stroke risk (In Chinese). 2019;  34. Yi L. Association of chromosome 9p21 single nucleotide polymorphism with ischemic stroke (In Chinese). 2015;  35. Feng CR. Effect of gene–gene and gene–environment interaction on the risk of first‐ever stroke and poststroke death. 2019. |
| Repeated publication=4 | 36. Yue XY. Chromosome 9p21.3 Variants Are Associated with Cerebral Infarction in Chinese Population. 2015;  37. Xiong L. The ANRIL Genetic Variation Is Associated with Atherothrombotic Stroke in Chinese Han Population in Northeast Sichuan (In Chinese). 2015;  38. Yi L. Association of 9p21 Single Nucleotide Polymorphism with Ischemic Stroke (In Chinese). 2015;  39. Xiong L. ANRIL genetic variation is associated with atherothrombotic stroke in Chinese Han population in Northeast Sichuan (In Chinese). 2015. |
| No clear baseline data=5 | 40. Da Silva CF. Ischemic Stroke and Genetic Variants: In Search of Association with Severity and Recurrence in a Brazilian Population. 2020;  41. Helgeland Ø. The Chromosome 9p21 CVD- and T2D-Associated Regions in a Norwegian Population (The HUNT2 Survey). 2015;  42. Karvanen J. The impact of newly identified loci on coronary heart disease, stroke and total mortality in the MORGAM prospective cohorts. 2009;  43. Matarin M. Whole Genome Analyses Suggest Ischemic Stroke and Heart Disease Share an Association With Polymorphisms on Chromosome 9p21. 2008;  44. Tragante V. The impact of susceptibility loci for coronary artery disease on other vascular domains and recurrence risk. 2013. |
| Not meet the purpose of the meta-analysis=46 | 45. An DW. Association of 9p21 single nucleotide polymorphisms with asymptomatic intracranial and extracranial arterial stenosis (In Chinese). 2018;  46. Liu J. Association between polymorphisms of chromosome 6, 7 and 9 with ischemic stroke in Han and Kazak nationalities (In Chinese). 2017;  47. Liu X. Association of GWAS-susceptibility loci with ischemic stroke recurrence in a Han Chinese population. 2020;  48. Cunnington MS. Chromosome 9p21 SNPs Associated with Multiple Disease Phenotypes Correlate with ANRIL Expression. 2010;  49. Liu H. The research on association of copy number variation in chromosome 9p21 region with atherothrombotic stroke in the Han Chinese population. 2017;  50. Liu Y. INK4/ARF Transcript Expression Is Associated with Chromosome 9p21 Variants Linked to Atherosclerosis. 2009;  51. ISGC. Genome-wide association study identifies a variant in HDAC9 associated with large vessel ischemic stroke. 2012;  52. Zhang K. Circulating lncRNA ANRIL in the Serum of Patients with Ischemic Stroke. 2019;  53. Liu R. Susceptible gene polymorphism in patients with three-vessel coronary artery disease. 2020;  54. Dichgans M. Shared genetic susceptibility to ischemic stroke and coronary artery disease – a genome-wide analysis of common variants. 2015;  55. Bozpolat A. The relationship between the prognosis of children with acute arterial stroke and polymorphisms of CDKN2B, HDAC9, NINJ2, NAA25 genes. 2019;  56. Huang T. LncRNA ANRIL regulates cell proliferation and migration via sponging miR-339-5p and regulating FRS2 expression in atherosclerosis. 2020;  57. Lasek-Bal A. The Association of SNPs Located in the CDKN2B-AS1 and LPA Genes With Carotid Artery Stenosis and Atherogenic Stroke. 2019;  58. Zeng WX. The correlation of serum long non-coding RNA ANRIL with risk factors, functional outcome, and prognosis in atrial fibrillation patients with ischemic stroke. 2020;  59. Jin W. The Single Nucleotide Polymorphisms of Chromosome 9p21 and CD147 Were Relevant with the Carotid Plaque Risk in Acute Cerebral Infarction Patients Among Chinese Han Population. 2020;  60. Haslacher H. 9p21.3 risk locus is associated with first-ever myocardial infarction in an Austrian cohort. 2014;  61. Zhao JK. Association of CDKN2B-AS1 rs1333049 with Brain Diseases: A Case-control Study and a Meta-analysis. 2017;  62. Schulz S. Single nucleotide polymorphisms in long noncoding RNA, ANRIL, are not associated with severe periodontitis but with adverse cardiovascular events among patients with cardiovascular disease. 2018;  63. Feng LJ. Circulating long noncoding RNA ANRIL downregulation correlates with increased risk, higher disease severity and elevated pro- inflammatory cytokines in patients with acute ischemic stroke. 2018;  64. Sun JL. Analysis on the Polymorphism of Chromosome Region 9p21 and the Susceptibility of Carotid Plaque. 2017;  65. Tian LB. 9p21 polymorphisms increase the risk of peripheral artery disease in the Han Chinese population. 2013;  66. Zhou SY. CDKN2B Methylation and Aortic Arch Calcification in Patients with Ischemic Stroke. 2017;  67. Zhou SY. CDKN2B methylation is associated with carotid artery calcification in ischemic stroke patients. 2016;  68. Wahlstrand B. The myocardial infarction associated CDKN2A/CDKN2B locus on chromosome 9p21 is associated with stroke independently of coronary events in patients with hypertension. 2009;  69. Dutta A. The coronary artery disease-associated 9p21 variant and later life 20-year survival to cohort extinction. 2014;  70. Hacke W. Commentary on a GWAS: HDAC9 and the risk for ischaemic stroke. 2012;  71. Sturiale CL. Association between the rs1333040 polymorphism on the chromosomal 9p21 locus and sporadic brain arteriovenous malformations. 2013;  72. Tsai PC. Additive Effect of ANRIL and BRAP Polymorphisms on Ankle-Brachial Index in a Taiwanese Population. 2012;  73. Ruigrok YM. From GWAS to the clinic: risk factors for intracranial aneurysms. 2012;  74. Adams H. Heritability and Genome-Wide Association Analyses of Intracranial Carotid Artery Calcification: The Rotterdam Study. 2016;  75. Sturiale CL. Association between Polymorphisms rs1333040 and rs7865618 of Chromosome 9p21 and Sporadic Brain Arteriovenous Malformations. 2013;  76. Hamrefors V. Smoking modifies the associated increased risk of future cardiovascular disease by genetic variation on chromosome 9p21. 2014;  77. Bendjilali N. Common variants on 9p21.3 are associated with brain arteriovenous malformations with accompanying arterial aneurysms. 2014;  78. Ärlestig L. Polymorphisms of the genes encoding CD40 and growth differentiation factor 15 and in the 9p21.3 region in patients with rheumatoid arthritis and cardiovascular disease. 2012;  79. Bai Y. Regulation of CARD8 Expression by ANRIL and Association of CARD8 SNP rs2043211 (p.C10X) with Ischemic Stroke. 2014;  80. Lin HF. Sex Differential Genetic Effect of Chromosome 9p21 on Subclinical Atherosclerosis. 2010;  81. Haver VG. The impact of coronary artery disease risk loci on ischemic heart failure severity and prognosis: association analysis in the Controlled ROsuvastatin multiNAtional trial in heart failure (CORONA). 2014;  82. Pott J. Genome-wide meta-analysis identifies novel loci of plaque burden in carotid artery. 2017;  83. Mehramiz M. Interaction between a variant of CDKN2A/B-gene with lifestyle factors in determining dyslipidemia and estimated cardiovascular risk: A step toward personalized nutrition. 2018;  84. Evaluation of Genomic Applications in Practice and Prevention (EGAPP) Working Group. Recommendations from the EGAPP Working Group: Genomic profiling to assess cardiovascular risk to improve cardiovascular health. 2010;  85. Kremer PH. Evaluation of genetic risk loci for intracranial aneurysms in sporadic arteriovenous malformations of the brain. 2015;  86. Musunuru K. Association of single nucleotide polymorphisms on chromosome 9p21.3 with platelet reactivity: a potential mechanism for increased vascular disease. 2010;  87. Low SK. Genome-wide association study for intracranial aneurysm in the Japanese population identifies three candidate susceptible loci and a functional genetic variant at EDNRA. 2012;  88. Larson MG. Framingham Heart Study 100K project: genome-wide associations for cardiovascular disease outcomes. 2007;  89. He P. The Diagnostic and Prognostic Value of Long Non-coding RNA ANRIL in Ischemic Stroke (In Chinese). 2019;  90. Anderson CD. Chromosome 9p21 In Ischemic Stroke: Population Structure and Meta-Analysis. 2010. |
