# Supplementary material for: Genetic association of ANRIL with susceptibility to Ischemic stroke: A comprehensive meta-analysis
Source: PLoS One. 2022 Jun 2;17(6):e0263459. doi: 10.1371/journal.pone.0263459 (PMC9162336; doi:10.1371/journal.pone.0263459)
Supplement: Attachment — Submitted filename: the second reply 1.14.docx [file pone.0263459.s004.docx]

Dear editors,

Thank you for these valuable comments, we have had a detailed answer to some problems. However, we had some difficult in dealing with other suggestions the reviewers mede. We hope that these faults will not affect the final decision the journal made. Please see this as follows:

*The title should have a clear, precise scientific meaning and should not contain a colon. Where possible, the title should be read as one concise sentence. Please re-write the title ensuring that it is informative and appropriate.*

Answer: the new title: Genetic association of ANRIL with susceptibility to Ischemic Stroke: a comprehensive meta-analysis

Reviewer #1:

*1. I am not sure if the 15 SNPs in ANRIL Gene are tagSNPs or not. Only tagSNPs that are unrelated with each other are informative, and it is better that the tagSNPs may cover this whole gene region.*

Answer: the objectives of the manuscript was all SNPs in ANRIL gene involving relationship to ischemic stroke, and aimed to investigate the role of ANRIL gene using SNPs as genetic marker in ischemic stroke risk. The SNPs were included in the meta-analysis only based on the SNP locus being studied in at least two articles. And so, it is not important whether the SNPs are tagSNPs.

*2. Multiple test correction should be conducted .*

Answer: We have discussed the focus in “Statistical analyses” section: “The false discovery rate (FDR) method by Benjamini–Hochberg was used for multiple testing correction”.

*3. Statiscial power should be calculaed with proper method to ensure less type 2 errors.*

Answer: In this meta-analysis, pooled ORs with corresponding 95% CI were calculated by the fixed-effects or the random-effects model based on three genetic models. This is a most common methods, and it is not important to calculate the statistical power again.

*3. subgroup analysis based on sex, age should be conducted.*

Answer: we have clearly described the proplem. Please see “Sub-population analyses were conducted for ethnicity, and meanwhile, subgroup analyses for IS subtype, age or gender (if available) were also performed” paragraph in “Statistical analyses” section.

*4. I would suggest collecting published GWAS data to redo the meta-analysis if it is possible.*

Answer: we only studied the SNPs in ANRIL gene in current manuscript. The GWAS data, case-only studies and family-based studies were not included because of different study design, as well as different analysis methods might be needed in meta-analysis based on GWAS data.

*5. For the genetic varients with significant association with the phenotype, deep discussion about the potential biological mechanisms involved in the phenotype is need. Especially, you had better see if this kind of SNP is causal or not.. Especially the potential causal effects for the SNPs with strong association signals should be explored.*

Answer: we have tried our best to make deep discussion on the potential biological mechanisms of these risk SNPs loci, however, some biological mechanisms for a few SNPs still is not available due to too few articles involving the loci.

*6, All the figures are not clear and need to be well tailored for publishing.*

Answer: we have revised the figures again.

*7,The language should be polished further.*

Answer: we have reviewed the language problems with the help of native English speaker.

Reviewer #2:

*In the manuscript entitled “The Research on Association of the ANRIL Gene with Ischemic Stroke: the Evidence From a Comprehensive Meta-Analysis”, the authors explored whether f the All the data used in the published GWASA papers should be included, if cannot, please state the reasons.*

*The following papers can be cited and followed for the meta-analytic procedures to improve the quality. (if the data is not enough available, at least DISCUSSION should be added as the LIMITATION of this study with enough citation to support the viewpoints):*

*I am not sure if the genetic polymorphism can be used for predict stroke based on a machine learning model. In the PRECISION MEDICINE era, deep learning or machine learning is a hot topic in classification and prediction of diseases based on biomarkers. The authors may discuss the possibility to use the genetic variants related to stroke for the prediction or early diagnosis of stroke. The authors may cite the following papers for discussion or follow the analytic procedures to construct machine learning prediction models.*

*I suggest one paragraph in the DISCUSSION section to elucidate the potential biological regulation mechanisms regarding how the genetic variant to affect the stroke outcome. The following papers clearly disclosed some genes indications whose abnormal expressions are mediated through mRNA modifications. The recent progress in N4-Acetylcytidine on RNA expression is also playing key role on the human diseases. I suggest the authors discussing this mRNA modifications/ N4-Acetylcytidine with their findings in the DISCUSSION section because the knowledge needs to be updated.*

Answer: the manuscript aimed to discuss the relationship of the ANRIL gene using SNPs as genetic marker in Ischemic stroke employing the meta analyses methods. The conclusions might be a association of the targeted gene with Ischemic stroke being detected or not, however, the results did not make us find some causal genes.

The GWAS data, case-only studies and family-based studies were not included because of different study design, as well as different analysis methods might be needed in meta-analysis based on GWAS data.

we have tried our best to make deep discussion on the potential biological mechanisms of these risk SNPs loci, however, some biological mechanisms for a few SNPs still is not available due to too few articles involving the loci.

Regards,

Hua Liu
